# Fall Prevention for Older Adults who use Wheelchairs and Scooters

**DOI:** 10.1093/geroni/igaf122.2628

**Published:** 2025-12-31

**Authors:** Laura Rice, Elizabeth Peterson, Toni Van Denend, Jacob Sosnoff, Deborah Backus

**Affiliations:** University of Illinois College of Applied Health Sciences, Urbana, Illinois, United States; University of Illinois Chicago, Chicago, Illinois, United States; University of Illinois Chicago, Chicago, Illinois, United States; University of Kansas Medical Center, Kansas City, Kansas, United States; Shepherd Center, Atlanta, Georgia, United States

## Abstract

Falls are common among the ∼1 million older adults who use wheelchairs and scooters (WC/S) full-time, especially people with Multiple Sclerosis (PwMS). Falls can result in injuries and concerns about falling (CaF) that limit active engagement in the community. To address these concerns, the individualized reduction of falls (iROLL) program was developed by an interdisciplinary team to meet the unique needs of people who use WC/S. iROLL applies a self-management approach to address topics including wheelchair skills, transfer activities, seated balance exercise, management of environmental factors, post-fall management, and maintenance of assistive technology. The program is delivered online. Participants asynchronously review pre-recorded lessons and synchronously meet with a physical or occupational therapist in groups of 2-5 participants. A pre/post, mixed methods study was used to examine the preliminary efficacy of iROLL. PwMS (n = 12) who use WC/S full time, age 62+/-12 years old, 92% female, participated in the study. After exposure to the six-week iROLL program, CaF significantly decreased (p < 0.01) and fall prevention and management knowledge significantly increased (p = 0.03). Participants reported a need for the program, valued peer learning, and attention to diverse influences on fall risk. This study is the first to examine the preliminary efficacy of an online fall prevention intervention for PwMS who use WC/S. While further evaluation is needed to examine iROLL’s impact on a larger and more diverse population, iROLL has good potential to meet a critical need for an underserved population of older adults aging with a disability.

